# Empowering breast cancer diagnosis and radiology practice: advances in artificial intelligence for contrast-enhanced mammography

**DOI:** 10.3389/fradi.2023.1326831

**Published:** 2024-01-05

**Authors:** Ketki K. Kinkar, Brandon K. K. Fields, Mary W. Yamashita, Bino A. Varghese

**Affiliations:** ^1^ Viterbi School of Engineering, University of Southern California, Los Angeles, CA, United States; ^2^ Department of Radiology & Biomedical Imaging, University of California, San Francisco, San Francisco, CA, United States; ^3^ Department of Radiology, Keck School of Medicine, University of Southern California, Los Angeles, CA, United States

**Keywords:** contrast enhanced mammography, radiomics, artificial intelligence, machine learning, deep learning, quantitative analysis, breast cancer detection

## Abstract

Artificial intelligence (AI) applications in breast imaging span a wide range of tasks including decision support, risk assessment, patient management, quality assessment, treatment response assessment and image enhancement. However, their integration into the clinical workflow has been slow due to the lack of a consensus on data quality, benchmarked robust implementation, and consensus-based guidelines to ensure standardization and generalization. Contrast-enhanced mammography (CEM) has improved sensitivity and specificity compared to current standards of breast cancer diagnostic imaging i.e., mammography (MG) and/or conventional ultrasound (US), with comparable accuracy to MRI (current diagnostic imaging benchmark), but at a much lower cost and higher throughput. This makes CEM an excellent tool for widespread breast lesion characterization for all women, including underserved and minority women. Underlining the critical need for early detection and accurate diagnosis of breast cancer, this review examines the limitations of conventional approaches and reveals how AI can help overcome them. The Methodical approaches, such as image processing, feature extraction, quantitative analysis, lesion classification, lesion segmentation, integration with clinical data, early detection, and screening support have been carefully analysed in recent studies addressing breast cancer detection and diagnosis. Recent guidelines described by Checklist for Artificial Intelligence in Medical Imaging (CLAIM) to establish a robust framework for rigorous evaluation and surveying has inspired the current review criteria.

## 1 Introduction

Breast cancer is the second most leading cause of cancer death in women globally (1), and early detection is crucial for improved prognosis (2–5). Digital Mammography (DM) is known to reduce breast cancer related deaths by 40%. However, among specific patients, heightened breast density poses a challenge in detecting early-stage small cancers, resulting in a higher rate of false positive callbacks and interval cancers (6, 7). Currently 43% of all women, 40–85 in age, have dense breasts warranting the need for additional screening beyond DM (8). In recent years, CEM has emerged as a potential option for offering improved sensitivity and specificity compared to current standards of breast cancer diagnostic imaging i.e., mammography (MG) and/or conventional ultrasound (US) (9, 10). CEM uses iodinated contrast to visualize tumour neovascularity and dual-energy DM to create a recombined or iodine image that highlights just the enhancing lesion in the breast (11, 12). CEM has comparable sensitivity to MRI with a much higher specificity, potentially at a much lower cost and higher throughput (13–15). As a natural progression, multiple studies report of the benefits of using CEM for the screening, diagnosis of breast cancers as a cost-effective and viable alternative to the current standards, particularly in women with dense breasts and at relatively higher risk of breast cancer.

From a technical standpoint, CEM employs anode and cathode components in x-ray tubes similar to conventional DM (16). It utilizes low and high-energy x-rays to highlight contrast agent-induced differences, aiding in tissue composition and distribution assessment (17, 18). Thus, CEM employs dual-energy method to produce high-resolution, low-energy digital mammogram images. These images are recombined to create a digitally subtracted image, which can be useful to identify vascularity of a particular lesion (12). Studies have suggested, low energy mammograms obtained as part of CEM protocols is comparable to conventional mammography (19, 20) and though with the added advantage of emphasizing regions of contrast enhancement (21). CEM is currently offered on five different systems across 4 vendors (11, 22). Although a general consensus on how to perform CEM has been followed, a standardized implementation has not been established. This is a difficult task considering the differences in system characteristics across vendors.

CEM has several drawbacks (11), including the risk of mild to severe hypersensitivity reactions due to iodinated contrast administration (23). Patients should be evaluated for a history of contrast material allergy. CEM radiation dose on average requires slightly higher radiation exposure when compared to conventional mammography in phantom studies, though do tend to fall beneath the 3 mGy threshold dose limit set by Mammography Quality Standards guidelines (24, 25). Furthermore, despite enhanced sensitivity of CEM, certain breast lesions may still be undetectable due to their location within the breast; supplementary breast MRI may be required if lesions are anticipated in these areas such as region near chest wall (26). Finally, due to use subtraction techniques, certain CEM-specific artifacts may be visible on the recombined image which likewise can obscure subtle lesion detection (27–29).

In recent years, there has been significant improvement in the field of Artificial Intelligence (AI) in healthcare, leading to better and more prompt treatment for patients. AI is a useful tool to supplement the abilities and knowledge of radiologists, oncologists, and pathologists, ultimately resulting in more precise and effective identification and treatment of breast cancer. The insights offered by Mongan et al. (30) regarding the importance of systematically presenting research findings resonate deeply within the academic and scientific community. Their assertion highlights that, beyond achieving optimal results in research, the meticulous and structured presentation of these findings is of paramount significance. These guidelines promote transparency, reproducibility, and the ability to generalize research findings. They standardize reporting, elevate research quality, and ensure clinical relevance, providing a shared foundation for researchers, reviewers, and clinicians to understand and assess deep learning studies effectively.

The goal of this review is to provide an overview of some of the basic ideas and advances in the use of for the detection of breast cancer using CEM. The limitations of conventional approaches will be addressed, as well as the ways in which these limitations can be removed using AI. Importantly, the review will include research that has looked at existing AI capabilities, as well as ideas on how these skills can be used in the clinical field.

## 2 Method

**Figure 1:**
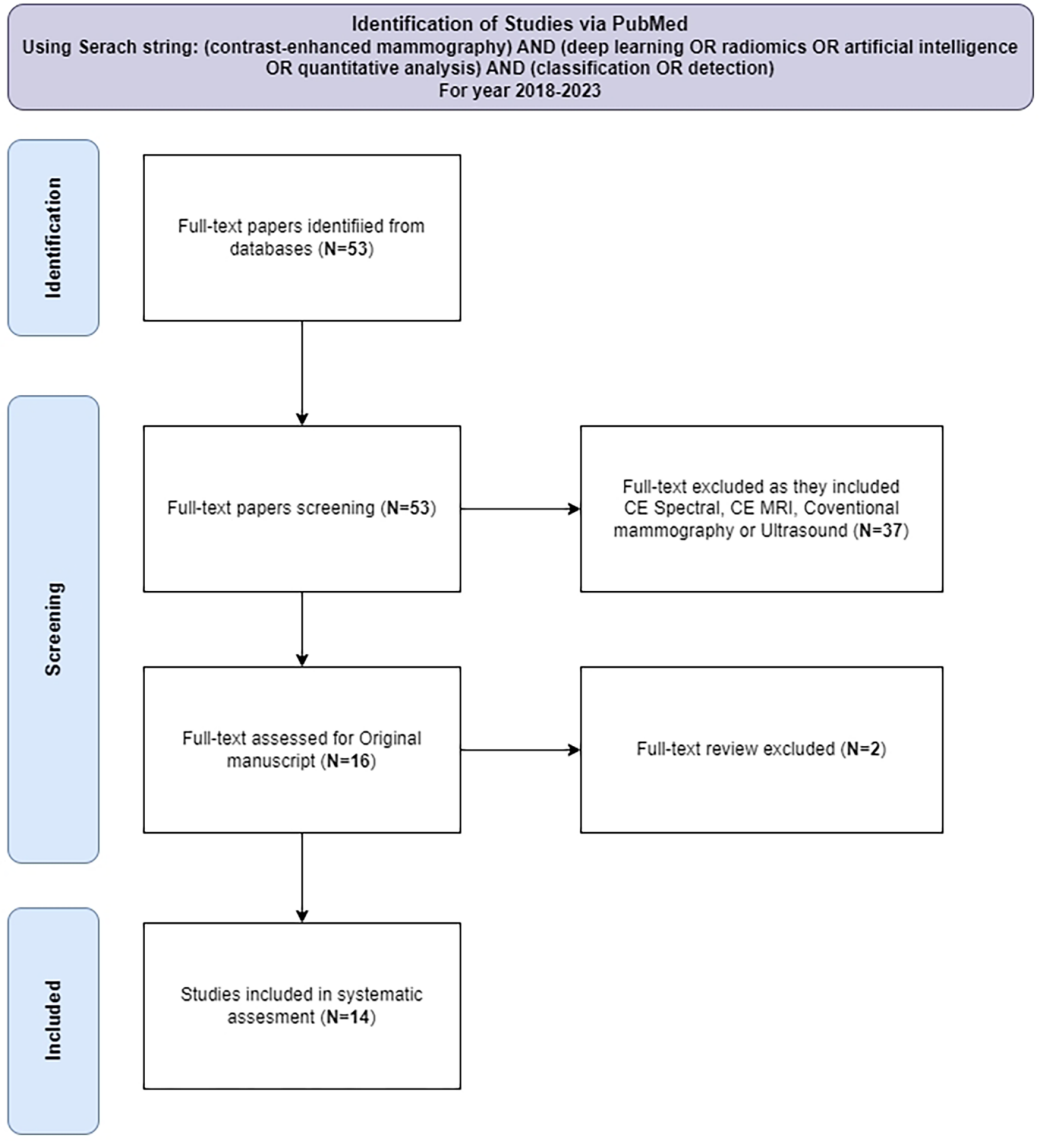
Diagram of systematic evaluation for article selection.

**Figure 2:**
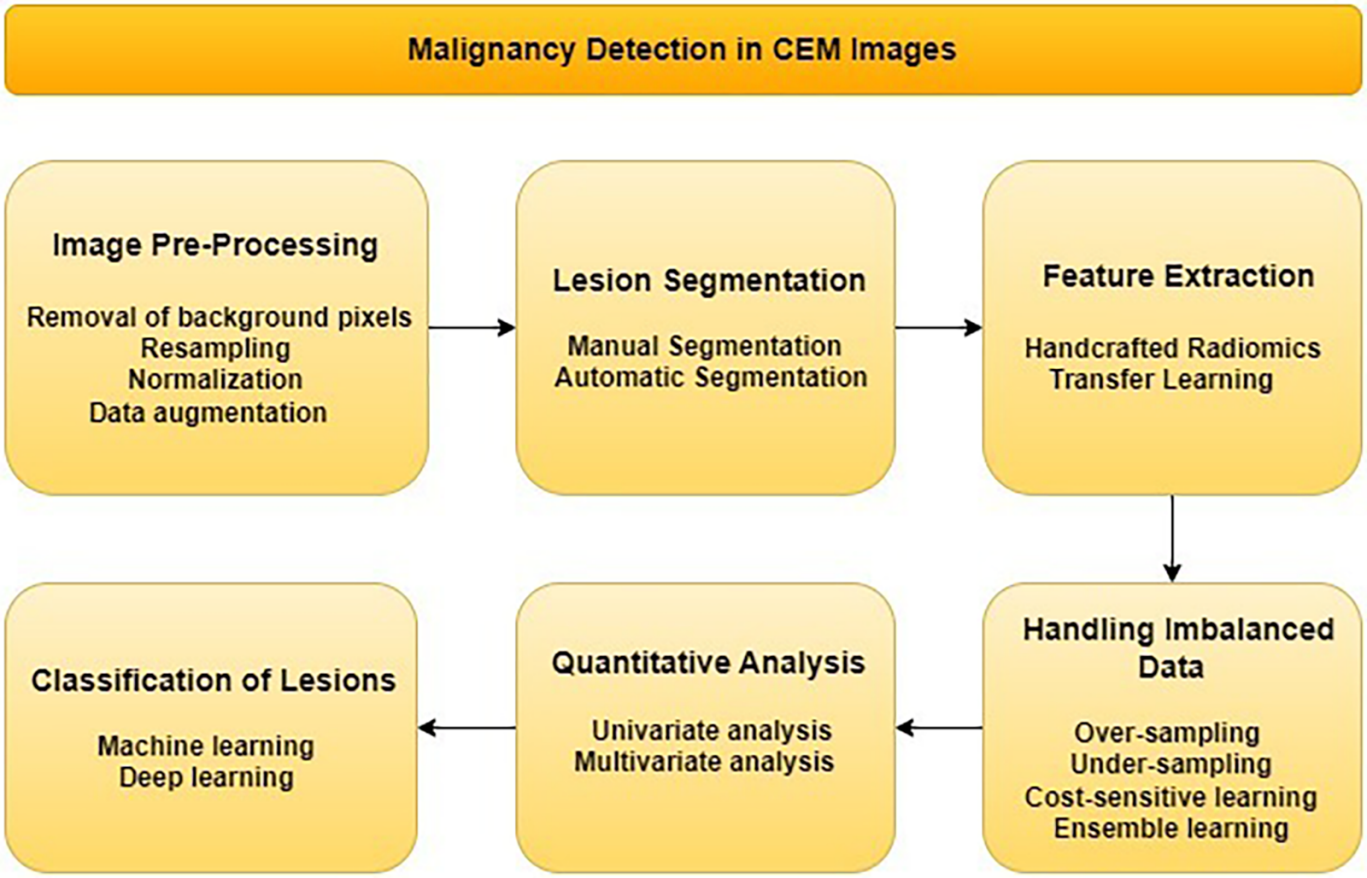
Flowchart for malignancy detection in CEM images.

The literature review was conducted on the use of contrast-enhanced mammography (CEM) and artificial intelligence (AI) techniques for predicting malignancy. PubMed database was searched for articles published between 1st January 2018 and 5th October, 2023, using a query: “(contrast-enhanced mammography) AND (deep learning OR radiomics OR artificial intelligence OR quantitative analysis) AND (classification OR detection)”. 53 articles that met this initial criteria were identified. Subsequently, each article was rigorously evaluated to ensure that it used CEM in conjunction with AI techniques to predict malignancy, resulting in a final selection of 14 articles. This rigorous selection process was documented in accordance with the PRISMA framework (31), which provides a transparent and structured methodology for article inclusion as shown in Figure 1. The following sections will discuss end-to-end malignancy detection pipelines using contrast-enhanced mammography. Flowchart of these methods is presented in Figure 2.

## 3 Image acquisition

The availability of CEM in commercial systems from vendors like GE Healthcare, Hologic, and Siemens Healthineers represents a significant advancement in breast imaging, as demonstrated in Table 1, with information sourced from Jeukens (32), Jochelson et al. (11). While optimal imaging parameters for CEM have not been extensively documented in published studies, there are a few generally accepted guidelines. Commonly, low-osmolarity iodine-based contrast in concentrations ranging from 300 mg/ml to 370 mg/ml at 1.5 ml/kg body weight (maximum 150 ml) is intravenously injected prior to image acquisition. Injection rates typically range from 2 to 3 ml per second (11). Among the reviewed studies, a total of 9 investigations made use of GE Healthcare systems, while 1 opted for Hologic systems and 3 opted to use data from both as mentioned in Table 2.

**Table 1:** Vendors for CEM imaging acquisition system.

Vendors/system characteristic	Low—energy imaging: anode and filter material and thickness	High—energy imaging: anode and filter material and thickness	Mean glandular dose	Total acquisition time
Vendor 1	Mo/Mo, Mo/Rh, Rh/Rh Mo (0.03 mm), Rh (0.025 mm)	Mo/AI + Cu, Rh/AI + Cu Al (0.3 mm), Cu(0.3 mm)	1.6–2.8 mGy	3–8 s
Vendor 2	Mo/Mo, Rh/Ag Mo (0.03 mm), Ag (0.03 mm)	Mo/Cu, Rh/Cu (0.25 mm)	0.7–2.3 mGy	2–5 s
Vendor 3	W/Rh, W/Ag (0.050 mm)	W/Cu (0.3 mm)	3.0 mGy	Less than 2 s
Vendor 4	W/Rh (0.050 mm)	W/Ti (1.0 mm)	–	15–22 s

Vendor 1 offers GE Healthcare's Senographe Essential and Senobright, Vendor 2 offers GE Healthcare Pristina and Senobright HD. Vendor 3 offers Hologic Selenia Dimensions and 3Dimensions I—View, and Vendor 4 offers Siemens Healthineers Mammomat Revelation Titanium CEM system. The low-energy tube voltage range for these vendors spans 26 to 34 kV, while the high-energy range extends from 45 to 49 kV. Essential anode and filter materials include silver (Ag), aluminium (Al), copper (Cu), molybdenum (Mo), rhodium (Rh), titanium (Ti), and tungsten (W). The data within this table is sourced from Jeukens (32), Jochelson at al (11).

**Table 2:** Review of existing work in AI for CEM imaging.

Research work	Methodology	CEM system	Data size	Benign/Malignant	Accuracy	Other metrics
Beuque et al. (33)	Mask RCNN with ResNet101	GE Healthcare	–	227/363 (External data set)	73%	AUC = 0.86 Sensitivity = 0.83 Specificity = 0.75
Wang et al. (34)	Least absolute shrinkage and selection operator (LASSO) logistic regression	GE Healthcare	226	101/125	88.2%	AUC = 0.96 Sensitivity = 0.90 Specificity = 0.93
Petrillo et al. (35)	Logistic Regression with LASSO	Hologic, USA and GE Healthcare	182	64/118	91.67%	Sensitivity = 0.90 Specificity = 0.92
Wang et al. (36)	Logistic regression	GE Healthcare	226	101/125	94.6%	AUC = 0.96 Sensitivity = 0.97 Specificity = 0.91
Fusco et al. (37)	Support Vector Machine	Hologic, USA and GE Healthcare	104	39/65	87%	AUC = 0.90 Sensitivity = 0.86 Specificity = 0.87
Wang et al. (38)	Least absolute shrinkage and selection operator (LASSO	GE Healthcare	223	101/122	–	AUC = 0.940
Sun et al. (39)	Least absolute shrinkage and selection operator (LASSO) regression	GE Healthcare	161	47/114	89.5%	AUC = 0.92 Sensitivity = 0.89 Specificity = 0.908
Miller et al. (40)	Penalized Linear Discriminant analysis	–	159	70/89	71.25%	AUC = 0.81 _ _Sensitivity = 0.56 Specificity = 0.75
Gao et al. (41)	ResNet along with Convolutional Neural Network (CNN)	Hologic, USA	49	23/26	89%	AUC = 0.91 Sensitivity = 0.93 Specificity = 0.86
Jailin et al. (42)	YOLOv5 with CSPDarknet as backbone	GE HealthCare, USA	7,443	3,739/3,704 estimated	90%	AUC = 0.964 FPR = 0.128
Zheng et al. (43)	RefineNet and Xception + Pyramid pooling (PPM)	GE Healthcare	1,802	493/1,309	87.6%	Sensitivity = 0.95 Specificity = 0.70
Savaridas et al. (44)	Artificial Neural Network (ANN)	Hologic and GE Healthcare	269	14/255	91.4%	AUC = 0.97 Sensitivity = 0.95 Specificity = 0.89
Chen et al. (45)	DenseNet 121 with Convolutional Neural Network (CNN)	GE Healthcare, USA	1,903	490/1,413	87.1%	AUC = 0.912 Sensitivity = 0.947 Specificity = 0.714
Qian et al. (46)	VGG16 with Convolutional Neural Network (CNN)	GE Senographe Essential	2,496	765/1,731	85%	AUC = 0.92 Sensitivity = 0.86 Specificity = 0.85

However, it is important to acknowledge that providing explicit details regarding image acquisition methods and the sources of ground truth data is essential for establishing a common platform for comparing existing studies. While the majority of researchers have embraced transparency and rigor in their research processes, there are exceptions where such critical information remains undisclosed. This underscores the importance of robust reporting standards and transparency within the scientific community to ensure the credibility and reproducibility of research findings. For example, information regarding vendor, model, and acquisition protocol must be provided in the publications. This is crucial since across the 4 major CEM vendors, there exist different strategies for performing dual-energy mammography, using different tube voltage ranges, anode materials, filter materials/thicknesses, and image reconstruction algorithms for creating the recombined CEM images. These differences can be a major source of inter-operator bias when using multivendor CEM within a multicenter study. Studies exploring harmonization/standardization strategies prior to using multivendor CEM data for multicenter studies are warranted.

## 4 Image pre-processing

Image preprocessing is crucial for models in contrast-enhanced imaging datasets, overcoming challenges like noise and artifacts. Steps like noise reduction, removal of background pixels, contrast enhancement, and data normalization improve image quality (47). Techniques like data augmentation, ROI extraction, and data balancing enhance model generalization and feature detection. This preprocessing standardizes datasets, enhancing performance and accuracy. Therefore, it is critical to establish image quality standards prior to inclusion into ML/DL applications for reliable pre-processing.

### 4.1 Removal of background pixels

The presence of artifacts in medical images can introduce confusion or even mimic lesions, potentially leading to unnecessary medical procedures. Therefore, the removal of these artifacts plays a pivotal role in enhancing the accuracy of diagnoses. Several techniques have been developed for artifact removal, including thresholding, clustering, graph-cut algorithms, and deep learning methods. Thresholding is particularly effective in addressing large and well-defined artifacts (47, 48). Clustering, on the other hand, groups similar pixels together to tackle artifact removal (48). Otsu's thresholding method has been applied in two notable studies (33, 43) for malignancy detection. In the case of (33), a two-step approach was employed, involving Contrast Limited Adaptive Histogram Equalization (CLAHE) before applying Otsu's thresholding. This preprocessing step, utilizing CLAHE, improved the image quality by mitigating issues related to uneven lighting conditions and varying contrast across different regions. Additionally, graph-cut algorithms provide another avenue for artifact removal, segmenting images based on pixel similarity (48). Deep learning techniques have also gained prominence, as they train neural networks to identify and subsequently remove artifacts (43). The choice of artifact removal technique hinges on the specific image characteristics and the desired outcome. Thresholding proves effective for larger and more distinct artifacts (49, 50) while clustering or graph-cut algorithms are better suited for smaller or grouped artifacts, offering a versatile array of tools to address artifact-related challenges in medical imaging.

### 4.2 Resampling

Resampling CEM images holds significance due to their high resolution, variable scan times, and diverse imaging protocols. Resampling is performed when there is a difference in the pixel resolution of an image. Image acquisition timing impacts appearance and generalization. Standardizing resolution and acquisition times enhances dataset consistency and diminishes model variance, ultimately reducing false negatives, thus improving model performance (51). Wang et al. (34) conducted a study that used data from two different centers and successfully standardized their dataset using resampling techniques. In study by Wang et al. (38) they performed resampling before feature extraction. Resampling CEM datasets with different resolutions in multi-source data scenarios is recommended, as it is likely to improve model performance.

### 4.3 Normalization

Given the wide variation in study protocols, acquisition systems, and contrast injection dosages, it is clear that these factors have a significant impact on the brightness, contrast, and color balance of CEM images. Normalization is performed when there is a difference in the pixel intensity values of the image. Certain image features, such as texture and contrast, are more sensitive to fluctuations in these parameters than others. Normalization techniques offer a critical solution to mitigate these sensitivities. By normalizing CEM images, the impact of variable brightness, contrast, and color balance is minimized (52). This, in turn, enhances the reliability and precision of feature extraction processes from CEM images. For instance, in a study by Zheng et al. (43) the researchers used data from three different sources. They used one source for training, and the other two for external testing. To ensure that the dataset was consistent, they used normalization. Qian et al. (46) enhanced CEM images by adjusting pixel values to improve contrast and highlight lesions and then performed min-max normalization. This normalization process was essential for harmonizing the diverse data sources and ensuring that the dataset was coherent and free of inconsistencies. Adopting these steps in studies is strongly recommended as they strengthen the reliability of their findings and conclusions and data integrity in multi-source studies.

### 4.4 Data augmentation

Being a relatively new technique, CEM studies face the challenges of being limited in size and imbalanced class distribution. These inherent characteristics pose a significant risk of overfitting, a scenario where the model becomes excessively attuned to the intricacies of the training data, hindering its ability to effectively generalize to unseen data. In response to this issue, data augmentation emerges as a valuable strategy. Data augmentation techniques, such as horizontal image mirroring, global intensity adjustments, realistic transformations of breast geometry (53), horizontal flipping, rotation, scaling, reducing size (54) and horizontal and vertical shifting have been effectively used in studies by Jailin et al. (42), Zheng et al. (43), Qian et al. (46). These techniques increase the diversity of the dataset, which improves the robustness of research findings.

### 4.5 Lesion segmentation

In the realm of radiomics, the extraction of features from lesion areas is a fundamental prerequisite. Achieving this necessitates the segmentation of lesions, a critical step in the process. Segmentation can be approached in two distinct ways.

#### 4.5.1 Manual segmentation

Manual segmentation remains a widely adopted and trusted technique for precisely delineating lesions in CEM images. This method involves the meticulous outlining of lesion boundaries. Typically executed by skilled radiologists. In several studies reviewed (34, 35, 37, 38, 40, 55) manual segmentation approach was the chosen method. This approach underscored the importance of detailed and careful delineation of lesion contours, taking into account both the CC and MLO views, thus emphasizing its role in achieving precision and accuracy in radiological assessments. It is crucial to recognize that manual segmentation, despite its accuracy and reliability, demands a substantial investment of time and effort. The involvement of skilled radiologists is paramount to its success. If radiologist availability is limited, a single radiologist may need to handle segmentation. However, for high accuracy and precision demands, involving multiple radiologists to review and segment the image could be advantageous. Also, although this labor-intensive process remains indispensable for not only its inherent precision but also its pivotal role in facilitating the development and evaluation of new automated segmentation methods.

#### 4.5.2 Automatic segmentation

Automatic segmentation is a rapidly developing field with the potential to improve the efficiency and practicality of CEM image analysis. Automatic segmentation methods leverage the power of deep learning models to develop a comprehensive understanding of lesion features in contrast-enhanced mammography (CEM) images, enabling them to autonomously outline lesion contours. Alternatively, whole-organ analysis, the analysis of the entire breast, can be performed instead of lesion-specific segmentation. Consequently, automatic segmentation methods have the potential to reduce analysis time and effort, while also enhancing the accuracy and reproducibility of segmentation outcomes. Numerous studies have contributed to the development and evaluation of automatic segmentation methods tailored for CEM images. By merging manual segmentation with artificial intelligence, Zheng et al. (43) introduced an approach that improved lesion segmentation accuracy and efficiency. Wang et al. (56) introduced methodology that emphasizes lesion localization, providing a user-friendly and efficient alternative to conventional segmentation techniques, specifically by applying a deep learning model to detect and localize lesions in CEM images. Meanwhile, Beuque et al. (33) utilized the Mask R-CNN model (57), a region-based deep learning model that is optimized for object detection and segmentation. Jailen et al. (42) employed the YOLO v5 model, a single-stage deep learning model that is faster and more generalized than Mask R-CNN. These examples exemplify the diversity of approaches within the realm of automatic segmentation, and highlight the different trade-offs between accuracy, speed, and generalization.

However, it is crucial to acknowledge that automatic segmentation methods are still in the process of development, and several significant challenges must be addressed before they can find widespread application in clinical practice. One pressing challenge pertains to the sensitivity of these methods to the quality of the training data. In cases where training data is noisy or incomplete, the model's ability to accurately grasp lesion features may be compromised. Additionally, the computational demands of automatic segmentation methods pose a formidable hurdle, especially in clinical settings characterized by limited resources.

## 5 Feature extraction

Feature extraction is a critical technique for training CEM model training, enhancing the accuracy, efficiency, and interpretability of deep learning models (58). Common techniques include shape features, texture features, and kinetic features. Shape features describe the shape of the lesion, texture features describe its brightness, contrast, and homogeneity, and kinetic features describe the changes in the lesion over time. It is the foundational step that lays the groundwork for subsequent model training. Feature extraction can be approached in two distinct ways, each bearing its own significance in the realm of medical imaging.

### 5.1 Handcrafted radiomics

The first method involves the extraction of handcrafted radiomics features from lesion regions, which have been meticulously annotated, segmented, or localized, as we previously discussed in the context of lesion segmentation. This approach, as observed in the reviewed studies, provides valuable insights into the characteristics of the lesion. These handcrafted features have been extracted using tools such as the PyRadiomics package and the Texture toolbox by MATLAB according to Image Biomarker Standardization Initiative (IBSI) (59), as elaborated in (33–37). Once these features are extracted, it becomes imperative to refine them to enhance data quality. This often involves normalization techniques to standardize the data and, importantly, assessing feature correlations using Spearman's coefficient. The subsequent crucial step to this feature extraction is feature selection (60) or the elimination of redundant features. The reviewed studies (34, 39, 55) have employed various methods for this purpose, such as interobserver agreement tests, Boruta's approach, Fisher criteria, maximum relevance minimum redundancy (mRMR), mutual information (MI), LASSO logistic regression (61), probability of error, pairwise correlations and average correlation (POE + ACC). Stratified 10-fold cross-validation is used in the XG Boost classifier to perform feature elimination (33). This process ensures that only the most informative and non-redundant features are retained for model training.

### 5.2 Transfer learning

Transfer learning is a valuable technique in deep learning pipelines for feature extraction. It utilizes pre-trained models to efficiently extract relevant features from new data, enhancing performance. This approach is particularly beneficial when working with small or noisy datasets, as it leverages knowledge learned from larger and more diverse datasets. This technique involves the use of pre-trained networks, such as Inception V3, CSP Darknet, Resnet, Xception, RetinaNet, VGG16 as observed in the reviewed studies (41–43, 45, 46, 55). Transfer learning offers computational efficiency and leverages higher-level features learned from extensive data, thus simplifying the feature extraction process from CEM images.

The choice between handcrafted radiomics and transfer learning hinges on the specific model being developed. Handcrafted radiomics requires lesion segmentation for feature extraction, while transfer learning allows for the utilization of either entire images or patches of lesions. This adaptability underscores the importance of selecting the most suitable approach based on the objectives and requirements of the model under consideration. In essence, feature extraction serves as the linchpin in the AI pipeline for malignancy detection and segmentation, determining the quality and effectiveness of subsequent model training.

## 6 Handling imbalanced data

Handling data imbalance is a critical step in the AI pipeline, often underestimated but profoundly influential in obtaining accurate outputs. Failure to balance data properly can result in false positives and false negatives, as data imbalance introduces bias toward the majority class, undermining the minority class. There are several common methods to tackle this problem:

### 6.1 Over-sampling

This approach involves generating synthetic samples for the minority class to bolster its representation in the training dataset. Techniques like SMOTE (Synthetic Minority Over-sampling Technique) (44) and ROSE (Random Over-Sampling Examples) can be employed for this purpose. For example, in studies (33, 35) the authors utilized Adaptive Synthetic Sampling (ADASYN).

### 6.2 Under-sampling

In contrast, under-sampling entails removing samples from the majority class to diminish its presence in the training dataset. Various techniques, such as random under-sampling and Tomek links, can be applied to implement under-sampling effectively. As indicated, the use of under-sampling may not be advisable for CEM Images due to the issue of limited data availability. In such cases, the removal of samples from the majority class could further exacerbate the data scarcity problem, potentially leading to inadequate representation of the majority class and negatively impacting the model's performance.

### 6.3 Cost-sensitive learning

This method assigns different costs to the misclassification of samples from different classes. By assigning a higher cost to the minority class, this approach compels the model to give more attention to it, often resulting in improved performance on imbalanced datasets as done in study (41).

### 6.4 Ensemble learning

Ensemble learning entails training multiple models on different subsets of the data and then averaging their predictions. This technique helps reduce model variance and enhances performance on imbalanced datasets.

These methods illustrate the versatility required to address data imbalance effectively and emphasize the importance of choosing the most suitable technique based on the specific dataset, as used by Gao et al. (41).

## 7 Quantitative analysis

Quantitative analysis of handcrafted features in CEM images encompasses the application of statistical and mathematical techniques to derive significant insights from the visual data. Following the extraction of these features from the lesion regions, it becomes imperative to subject the extracted features to rigorous measurement, quantification, and analysis before using these features for model training. Univariate and multivariate analysis represent two primary categories of quantitative methodologies extensively employed for the examination of handcrafted features within CEM images.

### 7.1 Univariate analysis

Univariate analysis is a fundamental statistical method focused on analyzing a single variable. It helps describe the variable's distribution, detect outliers, and identify trends, providing valuable insights into data characteristics. The non-parametric Wilcoxon-Mann-Whitney test is used for univariate analysis for handcrafted radiomics features in CEM research, as demonstrated in studies (35, 37). Its key benefits include not requiring specific data distribution assumptions, robustness against outliers, suitability for both ordinal and continuous data, and applicability to small sample sizes and non-normally distributed data. This is important because radiomics features are often non-normally distributed and can be susceptible to outliers. These attributes make it a valuable tool for comparing CEM radiomics features, ensuring robust and reliable research results. Another technique in study (37, 38) is the Intraclass Correlation Coefficient (ICC), which plays a vital role in univariate analysis for handcrafted radiomics features in CEM. The ICC assesses measurement reliability, identifies variability sources, aids in quality control, informs study design, facilitates feature reliability comparison, and determines clinical utility. By ensuring the consistency and trustworthiness of radiomics data, the ICC is essential for both research and clinical applications in CEM.

In univariate analysis, conducting Receiver Operating Characteristic (ROC) analysis and calculating the Youden index is a crucial step for determining the optimal cut-off value for each feature, also used by Wang et al. (36) to set optimal threshold for calculating accuracy and other parameters. This allows for the assessment of their discriminatory power and the identification of the point that maximizes sensitivity and specificity, which is essential for interpreting the performance of features, particularly in diagnostic or predictive modeling scenarios. Univariate analysis by Sun et al. (39) revealed that larger lesion sizes and rim or ripple artifacts were associated with more misclassifications of benign lesions and smaller lesion sizes were associated with more misclassifications of malignant lesions.

### 7.2 Multivariate analysis

Multivariate analysis involves the simultaneous examination of multiple variables, offering a powerful approach to uncover relationships among the features, classify data, and construct predictive models. It is a versatile tool for gaining deeper insights from complex datasets. Methods used for multivariate analysis of handcrafted radiomics features in CEM images include Principal Component Analysis (PCA) for dimensionality reduction, Linear Discriminant Analysis (LDA) (37, 62) for group discrimination, Logistic Regression (35) for binary outcome modeling, Random Forests for robust classification and regression, KNN (37) to handle outliers and non-linear relationships and Support Vector Machines (SVMs) for high-dimensional data analysis. These methods offer diverse approaches to extract insights from radiomics data, but their choice depends on research objectives and data characteristics. We recommend selecting the analytics technique that aligns with the specific criteria and research objectives. Multivariate analysis by Sun et al. (39) revealed that smaller lesion size and air trapping artifacts were associated with the misclassification of malignant lesions.

Our findings indicate that few studies have used handcrafted radiomics features, either independently or in conjunction with CEM images. Additionally, not all studies have conducted feature analysis. We strongly recommend incorporating these techniques into research endeavours. This would provide a more comprehensive understanding of the data, ultimately facilitating more effective model tuning during training.

## 8 Classification of lesions

After refining data from all the AI pipeline that we discussed in previous sections, the next important step in the AI pipeline for malignancy detection is to train a model to classify the data according to respective standards of ground truth. This can be done in two ways using machine learning techniques or using convolutional neural networks (CNNs).

### 8.1 Machine learning approach

Machine learning techniques play a vital role in malignancy detection from CEM images by distinguishing between malignant and benign lesions. In a review of 14 studies using CEM datasets as mentioned in Table 2, it was found that 7 of them used machine learning techniques for classification. Machine learning offers several advantages, including interpretability, which provides insights into how the model arrives at its outcomes. However, machine learning may not be the best choice for handling image data, such as CEM images, which are intricate and present challenges that traditional machine learning approaches may not effectively address. Machine learning is a highly suitable and effective choice for tasks where handcrafted features are used as the training data. Machine learning techniques can effectively harness the valuable insights extracted from handcrafted features to develop robust models for making informed predictions. Here is a comprehensive overview of the key methodologies:

#### 8.1.1 Logistic regression

Logistic regression is a binary classification technique known for its simplicity and effectiveness in distinguishing between two primary lesion categories. It plays a significant role in expediting cancer diagnosis. In study (34–36, 38, 39) it has been utilized alongside the Least Absolute Shrinkage and Selection Operator (LASSO), demonstrating good sensitivity for model outcomes. This combination of techniques provides a powerful approach for addressing classification challenges in medical research.

#### 8.1.2 Support vector machine

Support vector machine (SVM) is versatile tool that can be used for both binary and multi-class classification tasks. It is particularly well-suited for handling the complex high-dimensional radiomics data derived from CEM images, making it an invaluable asset in the pursuit of precise malignancy detection, as used by (37).

#### 8.1.3 Random forest

Random forest is a robust ensemble learning technique that combines multiple decision trees to improve prediction accuracy. Its innate resistance to noise and overfitting makes it dependable choices for navigating the complexities of radiomics data, emerging as steadfast allies when precision is of paramount concern as used by (36, 39).

#### 8.1.4 Linear discriminant analysis

Linear discriminant analysis (LDA), a supervised learning algorithm, can identify optimal linear feature combinations to discriminate between different data groups. Its utility is even more significant in the realm of high-dimensional radiomics data, where it facilitates the effective categorization of lesions as used by (37, 40).

### 8.2 CNN approach in deep learning

Convolutional Neural Network (CNN) is a deep learning technique that uses artificial neural networks to learn from data. Neural networks are inspired by the human brain and can learn complex patterns from data. CNN is well-suited for image analysis tasks, including malignancy detection in CEM images. CNN models can learn to identify subtle features in images that may be difficult or impossible for humans to see, making them very effective at distinguishing between malignant and benign lesions. In a review of 14 studies, 7 used CNNs for model training. 6 out of 7 studies used transfer learning with a pre-trained network as the backbone for their CNN architecture. Of these, 2 studies (33, 41) used the ResNet pre-trained network. ResNet (63) pre-trained network is a popular choice for training CNNs on medical datasets due to their depth, accuracy, and efficiency. They have been shown to be effective for a variety of medical image classification tasks and can be easily adapted to different datasets and tasks. In addition to ResNet, other pre-trained networks such as XceptionNet, CSPDarkNet, and Inception models (64) were also used in the reviewed studies.

Some studies using CNNs have not provided adequate information about key hyperparameters, such as learning rate schedule, optimization algorithm, minibatch size, dropout rates, and regularization parameters. Additionally, studies often fail to discuss why specific objective functions were chosen or how they align with the study's goals. We recommend researchers to define their criteria for selecting the best-performing model and clearly indicate when and how certain model parameters are restricted or frozen, especially in transfer learning scenarios. Adhering to these reporting standards would enhance transparency and reproducibility in CNN-based research for clinical and scientific purposes.

## 9 Cross validation

Cross-validation is essential for malignancy detection using CEM datasets because it prevents overfitting. CEM datasets are often small, making models more likely to overfit. Cross-validation assesses a model's ability to generalize by repeatedly testing it on different data subsets. It helps with model selection, hyperparameter tuning, and providing a robust performance estimate, ensuring reliable results in medical diagnosis. Commonly used CV methods encompass K-fold Cross-Validation, as indicated in (34, 39, 40, 43) which divides the data into subsets for rigorous evaluation. Stratified K-fold Cross-Validation is particularly beneficial for handling imbalanced datasets, ensuring that both malignant and benign cases are adequately represented. Leave-One-Out Cross-Validation, employed in (37, 41, 55) is suitable for smaller datasets but demands more computational resources due to its one-sample-at-a-time evaluation. Leave-P-Out Cross-Validation offers a middle ground for modest datasets. Repeated K-fold Cross-Validation enhances reliability by repeating the process multiple times. Nested Cross-Validation, although not cited in specific studies, plays a role in hyperparameter tuning. The choice of CV method hinges on factors like dataset size, class distribution, and research objectives, with Stratified K-fold commonly favored in CEM datasets to ensure equitable evaluation of model performance.

## 10 Integration with clinical data

The integration of clinical data with CEM datasets is a promising multi-modal approach for enhancing the accuracy and clinical utility of machine learning models for malignancy detection. This integration allows for a more holistic assessment of breast lesions by incorporating not only image-based features but also patient-specific clinical information. The extent to which this integration has been explored and implemented varies across studies. In addition to clinical data, some studies may also explore the combination of CEM with other imaging modalities, such as ultrasound, MRI, etc. These multi-modal approaches seek to leverage the complementary strengths of different data sources to improve the overall performance of malignancy detection models. The specific combination of modalities can vary depending on the research objectives and data availability. In the study by Miller et al. (40), they found that incorporating demographic and clinical information into their models led to a notably improved AUC-ROC compared to using only density images, contrast images, or the combination of density and contrast images. It is observed in study by Wang et al. (36), the inclusion of clinical features to the radiomics features for model training resulted in a significant increase in both accuracy and sensitivity.

In the research article reviewed in the Table 2, we observed that all of the studies used histopathology as their reference standard for obtaining final ground truth diagnosis results, with a follow-up period of 2 weeks to 2 years, depending on the study.

## 11 Future scope

In the current landscape of malignancy detection research, we have explored the various strategies employed by studies to attain their results. However, there exists a compelling scope in the realm of multimodal approaches, particularly considering the persistent challenge of data scarcity in medical image datasets. The incorporation of multimodal data holds the potential to revolutionize the field by augmenting the accuracy, sensitivity, and AUC of detection models. The rationale behind exploring multimodal approaches is rooted in the inherent strengths of deep learning. This robust tool enables the extraction of intricate features from one mode of data, which can subsequently be integrated with knowledge derived from another modality. By combining different sources of medical data, researchers can overcome the limitations posed by data scarcity and achieve a more comprehensive understanding of the underlying phenomena.

Multimodal data fusion can significantly improve malignancy detection models by leveraging the unique strengths of each modality. This approach can uncover hidden patterns and correlations, leading to improved patient outcomes and clinical decision-making. The future of malignancy detection research lies in strategic utilization of multimodal data, overcoming individual limitations and paving the way for more robust and accurate detection models. The integration of multimodal approaches holds the potential to redefine malignancy detection research.

## 12 Conclusion

In conclusion, advances in the field of Artificial Intelligence in Contrast-Enhanced Mammography (CEM) have occurred, holding enormous potential for changing breast cancer detection and radiology practice, however, largescale validation is warranted. This review study explored the many aspects of AI in CEM, including image processing, lesion segmentation, feature extraction, quantitative analysis, lesion classification, and integration with clinical data. The potential advantages are undeniably enormous. Timely identification and accurate diagnosis of breast abnormalities play a pivotal role in enhancing patient prognosis and minimizing unnecessary biopsy procedures. AI-powered CEM not only provides a more efficient and exact way of reaching these goals, but it also aids medical experts in to their decision-making processes. However, there is a lack of sufficient reliable labeled training data and handling variability between imaging systems, and protocols. Therefore, while AI analysis shows promise for improving CEM diagnosis, larger studies assessing its clinical value and real-world effectiveness are required. For such studies to be designed and implemented, it is critical that researchers, doctors, and technologists continue to interact and push the bounds of artificial intelligence in CEM. The synergistic partnership between AI and medical practitioners has the potential to usher in a new era of breast cancer diagnosis that prioritizes precision and efficiency. As a result, we can make great progress in lowering the burden of breast cancer and improving the lives of individuals afflicted by it.
